# Novel Therapeutic Approaches for Various Cancer Types Using a Modified Sleeping Beauty-Based Gene Delivery System

**DOI:** 10.1371/journal.pone.0086324

**Published:** 2014-01-21

**Authors:** In-Sun Hong, Hwa-Yong Lee, Hyun-Pyo Kim

**Affiliations:** 1 Adult Stem cell Research Center, Seoul National University, Seoul, Republic of Korea; 2 Department of Veterinary Public Health, Laboratory of Stem Cell and Tumor Biology, Seoul National University, Seoul, Republic of Korea; 3 Department of Biomedical Science, Jungwon University, Chungbuk, Korea; University of Nantes, France

## Abstract

Successful gene therapy largely depends on the selective introduction of therapeutic genes into the appropriate target cancer cells. One of the most effective and promising approaches for targeting tumor tissue during gene delivery is the use of viral vectors, which allow for high efficiency gene delivery. However, the use of viral vectors is not without risks and safety concerns, such as toxicities, a host immune response towards the viral antigens or potential viral recombination into the host's chromosome; these risks limit the clinical application of viral vectors. The Sleeping Beauty (SB) transposon-based system is an attractive, non-viral alternative to viral delivery systems. SB may be less immunogenic than the viral vector system due to its lack of viral sequences. The SB-based gene delivery system can stably integrate into the host cell genome to produce the therapeutic gene product over the lifetime of a cell. However, when compared to viral vectors, the non-viral SB-based gene delivery system still has limited therapeutic efficacy due to the lack of long-lasting gene expression potential and tumor cell specific gene transfer ability. These limitations could be overcome by modifying the SB system through the introduction of the hTERT promoter and the SV40 enhancer. In this study, a modified SB delivery system, under control of the hTERT promoter in conjunction with the SV40 enhancer, was able to successfully transfer the suicide gene (HSV-TK) into multiple types of cancer cells. The modified SB transfected cancer cells exhibited a significantly increased cancer cell specific death rate. These data suggest that our modified SB-based gene delivery system can be used as a safe and efficient tool for cancer cell specific therapeutic gene transfer and stable long-term expression.

## Introduction

Gene-directed enzyme prodrug therapy (GDEPT) is one of the promising alternatives to conventional chemotherapy; GDEPT minimizes systemic toxicities through the introduction of catalytic enzymes that convert low- or non-toxic prodrugs into toxic metabolites in tumor cells [1]. This therapeutic system comprises of inactive low- or non-toxic prodrugs and a gene encoding an enzyme [2]. After genetically modifying the tumor cells to express such enzymes and the systemic administration of the prodrug, the prodrug is locally converted by the enzyme into toxic metabolites, leading to the selective killing of the tumor cells. Because the toxic metabolite is only produced and released in the local tumor site where the gene is delivered, resulting in a greatly reduced circulating concentration of the free toxic drug, this therapeutic system is called local chemotherapy. There are several genes encoding prodrug-activating enzymes. Among them, the most common gene is Herpes Simplex Virus-1 Thymidine Kinase (HSV-TK), a well characterized suicide gene that can be isolated from the Herpes simplex virus or *E. coli* [3], [4]. HSV-TK converts the systemically administered prodrug gancyclovir (GCV) into a toxic metabolite that kills cancer cells [5]. This combination method has been successfully applied to many clinical areas, such as gene therapy for cancer treatment [6] and graft-versus-host disease (GVHD) [7].

Successful GDEPT largely depends on the selective introduction of the suicide gene into the appropriate target cancer cells. One of the most effective and promising approaches for gene delivery into the target tumor tissue is the use of viral vectors, which allow for high efficiency gene transfer [8], [9]. However, these vectors currently have risks and safety concerns, such as toxicities, host immune responses towards the viral antigens or potential viral recombination into the host's chromosomes, which limit their clinical application [10]. Another problem with the use of viral vectors for gene therapy is that viral vector preparation is laborious and expensive [11], [12] Therefore, a better alternative may be to develop a gene delivery method that would direct therapeutic agents to appropriate sites and constitutively express therapeutic genes within or near the tumor site without these limitations.

The transposon-based system is an attractive, non-viral alternative to the viral delivery systems. Transposons are discrete segments of DNA that have the inherent ability to move from site to site and can replicate themselves within the genome using the host cell's organelles and other machinery. However, when compared to viral vectors, transposons are not infectious, and their activities are therefore confined to intracellular compartments with specific functions. In invertebrates, several different types of endogenous transposons have been extensively used, such as the Tc1 mariner-like element in *Caenorhabditis elegans* and the P element in *Drosophila* [13], [14]. Unfortunately, no such active and endogenous transposons are available in vertebrates due to the accumulated mutations within the transposon sequence [15]. One approach to overcome this problem in vertebrates was the molecular reconstruction of a genetically active vertebrate Tc1/mariner-type transposable element called Sleeping Beauty (SB) from the ancient “dead” transposon fossils found in fish genomes [16]. SB shows efficient transpositional activity in mouse embryonic stem (ES) cells [17], mouse somatic tissue [18], and mouse germ line [19]. In addition, SB has shown tremendous success as an efficient gene delivery vehicle in mouse models of human disease [20]–[23]. In previous studies, song et al. found that SB transposon mediate suicide gene expression in hepatocellular carcinoma causing apoptosis [24] and telomerase gene transfer to protect against chemicals (t-BH, CCl4, or d-GalN )- induced acute cellular injury [25].

However, when compared to viral vectors, the non-viral SB based gene delivery system still has limited therapeutic efficacy due to the lack of long-lasting gene expression and cancer cell specific gene transfer ability. The hTERT gene is frequently reactivated in approximately 90% of immortalized human cells and cancer cells of various origins [26]–[28]. hTERT promoter has been extensively used in targeted cancer gene therapy [29]–[31]. Additionally, the SV40 enhancer has been extensively used to improve the activity of promoters to promote long-lasting gene expression [32]. Thus, to increase the promoter strength while maintaining tissue specificity, we used a recombinant SV40 enhancer containing a tumor-specific hTERT promoter [33]. While song et al. have reported an association between modified SB and suppression of tumor growth [24], their study have been confined to those of single hepatocellular carcinoma cell line (Hep3B) in which univeral antitumor effects in various cancer cell types are lacking. Similarly, although tumor suppressive effects of modified SB have been suggested, these effects are confirmed by a single in vitro assay (ATP cell viability assay) in which a more comprehensive profile of the antitumor effects of modified SB is still lacking [24]. Therefore, in the present study, the modified SB-based gene delivery system was used to transfer the suicide gene (HSV-TK) into multiple types of cancer cells, including H358 (lung cancer), H1299 (lung cancer), PC3 (prostate cancer), DU145 (prostate cancer), and OVCAR3 (ovarian cancer) cells with various experimental approaches including TUNEL assay, cell viability assay, FACS analysis, and in vivo experiment. These data suggest that modified SB-based gene delivery system can be used as a safe and efficient tool for cancer cell specific therapeutic gene transfer and stable long-term expression.

## Materials and Methods

### Cell culture

Human normal fibroblast, WI-38 and IMR-90 cells were maintained in DMEM (GIBCO BRL, Germany) supplemented with 10% fatal calf serum (FCS) (HyClone, Logan, USA), penicillin (50 units/ml), and streptomycin (50 ug/ml) in the presence of 5% CO_2_. H358 (lung cancer), H1299 (lung cancer), PC3 (prostate cancer), DU145 (prostate cancer), and OVCAR3 (ovarian cancer) were grown in RPMI1640 supplemented with 10% FCS, penicillin (50 units/ml), and streptomycin (50 ug/ml) in 5% CO2. All cell lines were obtained from Korea Cell Line Bank (Seoul, Korea).

### Animal experiments

Lung cancer cells (H358), prostate cancer cell line (DU-145), and ovarian cancer cells (OVCAR3) were harvested by trypsinization, and 1×10^5^ viable cells (as determined by trypan blue exclusion) in a total volume of 200 µl were injected subcutaneously into 6- to 10-week-old, NOD/SCID mice. Two days following tumor seeding, animals were intravenously injected via tail veins with 100 mg/kg gancyclovir (GCV) along with either 25 µg of empty plasmid (pT. hTp. Con) or modified SB system (pT.hTp.HSV-tk.Con). Mice were sacrificed 28 days after tumor injection, and the effect of modified SB system on tumor growth was evaluated by measuring tumor size. To minimize suffering, all surgical procedure was performed under sodium pentobarbital anesthesia. Animal experiments were approved by the ethics committee for Animal Experiments of Jungwon University (Permit Number: 2013-0610).

### RT-PCR analysis

Total RNA was isolated from each cells using the Absolutely RNA Microprep kit (Stratagene, La Jolla, USA) according to the manufacturer's protocol and treated with DNase I to prevent genomic DNA contamination. cDNA synthesis was performed with 1 ug of RNA using the SuperScript III reverse transcriptase (Invitrogen, Calsbad, USA) with Oligo(dT) primer (Promega, Madison, USA) to a final volume of 20 ul. 1 ul aliquots of cDNA were used to amplify the cDNA in 20 ul of total reaction. PCR conditions for amplification were: 94°C for 5 min; 30 cycles at 94°C for 1 min, at 55°C for 1 min, and at 72°C for 90 sec; and finally, 72°C for 10 min). hTR cDNA was amplified with hTR specific primer set (5′-tttgtctaaccctaactgagaagg-3′ as forward and 5′-tgtgagccgagtcctgggtgcacg-3′ as reverse). hTERT cDNA was amplified with forward primer (5′-cggaagagtctggagcaa-3′) and reverse primer (5′-ggatgaagcggagtctgga-3′). Differences in expression from each sample were normalized to the GAPDH (forward 5′-aacgagcggttccgatgccctgag-3′; reverse 5′-tgtcgccttcaccgttccagt t-3′). The PCR products were separated by 2% agarose gel electrophoresis containing 0.5 µg/ml of ethidium bromide.

### Construction of plasmids

**Figure 1:**
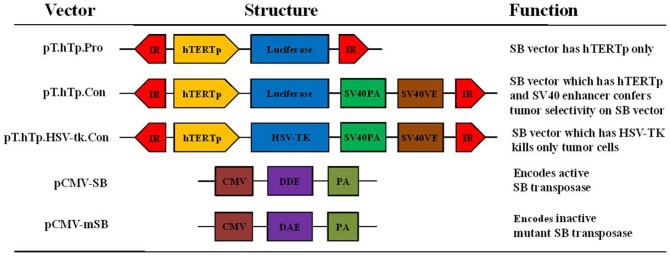
Schematic representation of the Sleeping Beauty transposon including the hTERT promoter and SV40 enhancer used in our studies. To construct the transposon plasmid, the pGL3-hT-Con and pGL3-hT-TK-Con plasmids were cut with KpnI and BamHI; the ends of the luciferase cassette and the HSV-TK cassette were filled in using DNA polymerase I and blunt end ligated into the pT-MCS vector, resulting in the pT.hTp.Con and pT.hTp.HSV-tk.Con vectors, respectively. The pT.hTp.nori.Con plasmid was constructed from the pTnori plasmid through the insertion of the human telomerase promoter (hTERTp) and the SV40 enhancer. The SV40 promoter was replaced with hTERTp by digesting the pTnori plasmid with SexAI and BglII. The SV40 enhancer was inserted between the BsmI and BamHI sites of pTnori. The hTERT promoter and the SV40 enhancer region were amplified by PCR from the pGL3-hT-Con vector using Ex-Taq polymerase.

Plasmids for the Sleeping Beauty-transposon system, pCMV-SB and pCMV-mSB (a transposase having a missense mutation in its C-terminal Asp-Asp-Glu motif) were kindly provided by Dr. Joonseok Song (University of Pittsburgh) [25]. To construct the transposon plasmid, pGL3-hT-Con and pGL3-hT-TK-Con plasmids were cut with KpnI and BamHI restriction enzymes. A luciferase and the HSV-TK gene were ligated with pT-MCS vector, which gave rise to pT.hTp.Con and pT.hTp.HSV-tk.Con vectors respectively (Fig. 1). The hTERT promoter and the SV40 enhancer region were amplified by PCR using Ex Taq DNA polymerase (Takara, Shiga, Japan) and subcloned into the pGL3-hT-Con vector. The hTERT promoter forward (5′-accaggtagtggattcgcgggcacaga-3′) and reverse (5′-agatctagggcttcccacgtgcgcag-3′) primers, and the SV40E forward (5′-gcattcgatggagcgg-3′) and reverse (5′-ggatccgctgtggaatg-3′) primers were used. PCR was performed for 35 cycles at 94°C for 1 min, at 60°C for 1 min, and at 72°C for 1 min.

### Luciferase assay

Luciferase assays were carried out using Luciferase Assay System (Promega, Madison, USA) and AutoLumat LB953 luminometer (Berthold, Wildbad, Germany) according to the manufacturer's protocols. In brief, 3×10^4^ cells seeded in a 16-mm tissue culture dish were transfected with 1 ug of luciferase reporter plasmids and 0.25 ug of pSV-β-galactosidase control plasmid vector. Cell lysates were prepared 48 hours after transfection by adding 100 ul of reporter lysis buffer and their luciferase activities were measured. The pGL3-Promoter plasmid containing SV40 promoter was used as a positive control. The luciferase activity of the pGL3-Promoter plasmid in each cell line was considered to be 1, and the relative luciferase activity was calculated. All luciferase assays were performed in triplicate.

### Plasmid transfection with GCV treatment in vitro

Cancer and normal cells (10^5^ cells per dish) were plated in 35-mm culture dishes prior to transfection, and they were transfected with pCMV-SB or pCMV-mSB plasmid with pT.hTp.HSV-TK.Con plasmid using Lipofectamine 2000 transfection reagent (Invitrogen, Carlsbad, USA). After incubation at 37°C for 3 days, 2 ml of media containing various concentrations of Ganciclovir (GCV; InvivoGen, San Diego, USA) were added to the cells. All of the data shown in this report were obtained from at least three independent experiments.

### TUNEL assay

DNA strand breaks in apoptotic cells were measured by the TUNEL assay using the In-situ Detection Kit (Roche Molecular Biochemicals, Germany). Samples were fixed with 4% paraformaldehyde in PBS for 15 min and incubated in a 0.1% ice-cold Triton X-100 solution for permeabilization for 10 min according to the manufacturer's instructions. Cell were then washed 3 times with PBS and reacted with 50 µl of the TUNEL reaction mixture at 37°C for 60 min in a dark, humidified chamber. Cells were washed three times in PBS. Under fluorescence microscopy, the number of TUNEL-positive cells was counted.

### Apoptosis assay

The apoptotic rates in normal and cancer cells were measured by an Annexin V-FITC Kit (Invitrogen, Carlsbad, USA). Briefly, the pT.hTp.HSV-TK.Con and pCMV-SB transfected cells were treated with GCV (50 ug/ml, 3 hr at 37°C) after 10 days of transfection. Cells were harvested and rinsed with a 1× annexin-binding buffer and then resuspended with 100 ul of a 1× annexin-binding buffer. After adding 5 ul of FITC annexin V and 1 ul of propidium iodide (PI; 100 ug/ml), cells were incubated at room temperature for 15 minutes in the dark. The proportion of viable cells were analyzed by FACScalibur flow cytometer (Becton Dickinson, San Jose, CA) and quantified by Flowjo software (Tree Star Inc., Ashland, USA).

### Cytotoxicity assay

A number of 5×10^3^ pT.hTp.HSV-TK.Con and pCMV-SB transfected cells were inoculated into 96-well plate. The plate was then incubated at 37°C and 5% CO2 for 24 hours. GCV was added at the concentration level of 10, 30, 50 ug/ml in triplicate for each concentration level. The cells were then incubated for 3 hours and were added with MTT, where the cells were cultured for another 4 hours. Supernatant was then removed and DMSO was added to dissolve the MTT formazan crystals. OD value at 570 nm was detected by a microplate reader. Cell survival rate was calculated by: survival rate (%) = A/B×100 (A: OD value of pT.hTp.HSV-TK.Con and pCMV-SB transfected cells; B: OD value of pT.hTp.Con and pCMV-SB). All experiments were performed in triplicate. Survival curve was generated with mean viabilities of each cell lines as ordinate axis and the concentrations of GCV as abscissa axis

### Statistical analysis

Each set of experiments was performed in duplicate or triplicate. Results from each experiment replicate were presented as the mean ± SD. Data were assessed by one-way analysis of variance (ANOVA) and significant results were further assessed by Tukey's multiple comparison tests. Statistical significance was defined at a P level of <0.05.

## Results

### hTERT is a promising target for the SB-mediated gene therapy

**Figure 2:**
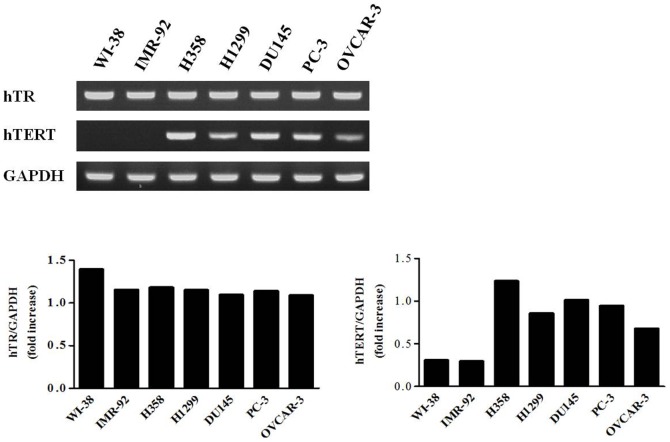
Comparison of the hTR and hTERT expression profiles of multiple cancer cell lines and normal fibroblasts. hTR and hTERT expression levels in various cancer cell lines and normal fibroblasts were determined using RT-PCR. Total RNA was amplified using hTR and hTERT specific primers (top and middle, respectively). GAPDH expression was used as an internal control for the normalization of hTR and hTERT expression. hTR was constitutively expressed in both the fibroblasts and the cancer cell lines. The expression of hTERT was detected in all cancer cells, whereas it was not detected in the two fibroblast cell lines. Lung cancer cell lines (H358 and H1299); prostate cancer cell lines (PC3 and DU145); ovarian cancer cell line (OVCAR3); fibroblast cell lines (WI-38 and IMR-90).

Successful gene therapy largely depends on the selective expression of a suicide gene in appropriate target cancer cells, but not in the normal surrounding cells. The human telomerase reverse transcriptase (hTERT) gene, which encodes the catalytic subunit of telomerase, is highly expressed in embryonic stem cells and is progressively down-regulated during differentiation, resulting in complete silencing in fully differentiated somatic cells. hTERT is frequently reactivated in approximately 90% of immortalized human cells and cancer cells of various origins [26]–[28]. Therefore, it can be a promising target for gene therapy in a variety of tumor types. First, we examined the expression profiles of the human telomerase RNA components (hTR) and hTERT using RT-PCR in fibroblasts and various cancer cell lines. hTR was constitutively expressed in both the fibroblasts and the cancer cell lines, which were consistent with previous observations [34]. The expression of hTERT was detected in all cancer cells, including two lung cancer cell lines (H358 and H1299), two prostate cancer cell lines (PC3 and DU145), and an ovarian cancer cell line (OVCAR3), but it was not detected in the two fibroblast cell lines (WI-38 and IMR-90) (Fig. 2). Therefore, expression of hTERT appeared to be tightly connected to tumor development, and hTERT could be a promising target for the SB-mediated gene therapy of the various cancer types.

### Tumor specific activation of the hTERT promoter by the SV40 enhancer

**Figure 3:**
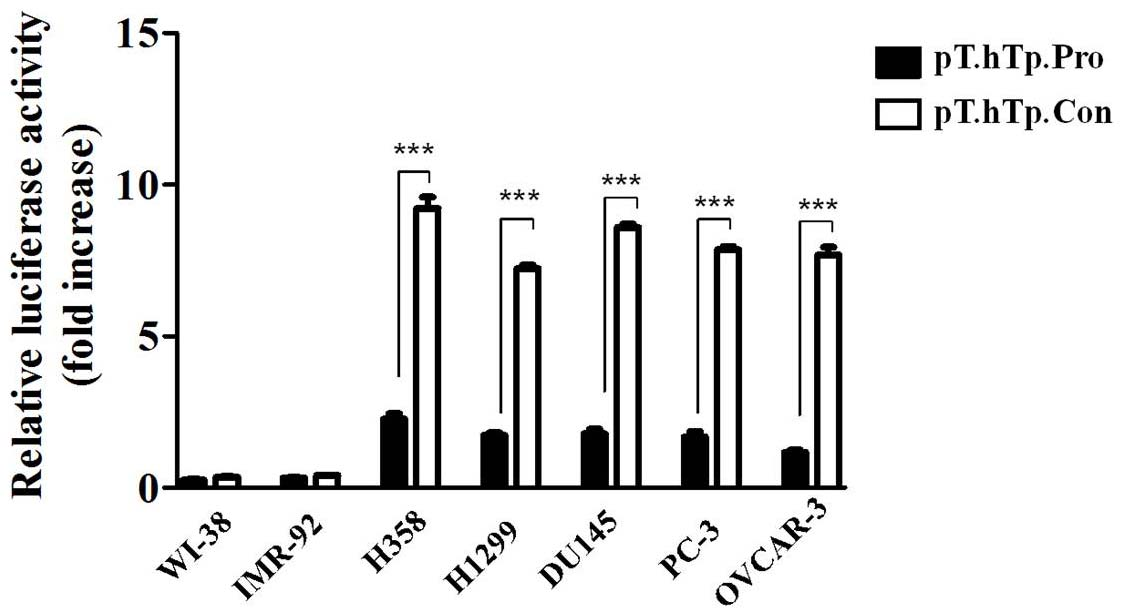
Comparison of the hTERT-mediated transcriptional activity of various cancer cell lines and normal fibroblasts with or without the SV40 enhancer and SV40 PolyA. Following the transfection of either the pT-hTp-Con plasmid (with SV40 enhancer and SV40 PolyA) or the pT-hTp-Pro plasmid (without SV40 enhancer and SV40 PolyA), the luciferase activity was detected and compared in normal and cancer cell lines. In telomerase positive cancer cell lines, the SV40 enhancer and SV40 PolyA activated the hTERT gene promoter by at least 7-fold compared to the hTERT gene promoter activity without these sequences. Relative luciferase activity was standardized to the transfection of the control pGL3-promotor plasmid. Lung cancer cell lines (H358 and H1299); prostate cancer cell lines (PC3 and DU145); ovarian cancer cell line (OVCAR3); fibroblast cell lines (WI-38 and IMR-90). The results are shown as the mean ± SD from three independent experiments. * P<0.05, ** P<0.01, and *** P<0.001.

Because the normal or natural promoter was not strong enough to induce efficient expression of the therapeutic genes in certain tumor cells, a powerful tumor-specific promoter is essential to achieve successful long-lasting therapeutic gene expression. The Simian virus 40 (SV40) enhancer has been extensively used to improve the activity of promoters [32] and the 3 poly(A) tail is important for the stability and translation mRNA [35]. Thus, to increase the promoter strength while maintaining tissue specificity, we constructed a recombinant hTERT promoter that contained the SV40 enhancer and SV40 PolyA (Simian virus 40 PolyA, also called PolyA). The SV40 enhancer sequence was amplified and inserted into the multiple clone site (MCS) downstream of the hTERT promoter. The transcription activity following transfection with the pT-hTp-Con plasmid (with SV40 enhancer and SV40 PolyA) or the pT-hTp-Pro plasmid (without SV40 enhancer and SV40 PolyA) was detected and compared in the normal and cancer cell lines, and the effect of the SV40 enhancer on the activity of the tumor specific hTERT promoter was evaluated. As shown in Fig. 3, the pT-hTp-Con plasmid, which contains the SV40 enhancer and SV40 PolyA, showed a significant increase in transcript activity in the telomerase positive cancer cell lines, whereas there was no change in the two normal fibroblast cell lines. These results showed that the hTERT promoter exhibited relatively high transcriptional activities in most of the tumor cell lines while exhibiting little activity in the normal fibroblast cell lines, and the SV40 enhancer and SV40 PolyA significantly increased the activity of the hTERT promoter exclusively in the cancer cell lines.

### Long-term sustainability of the modified SB delivery system in various types of cancer cells

**Figure 4:**
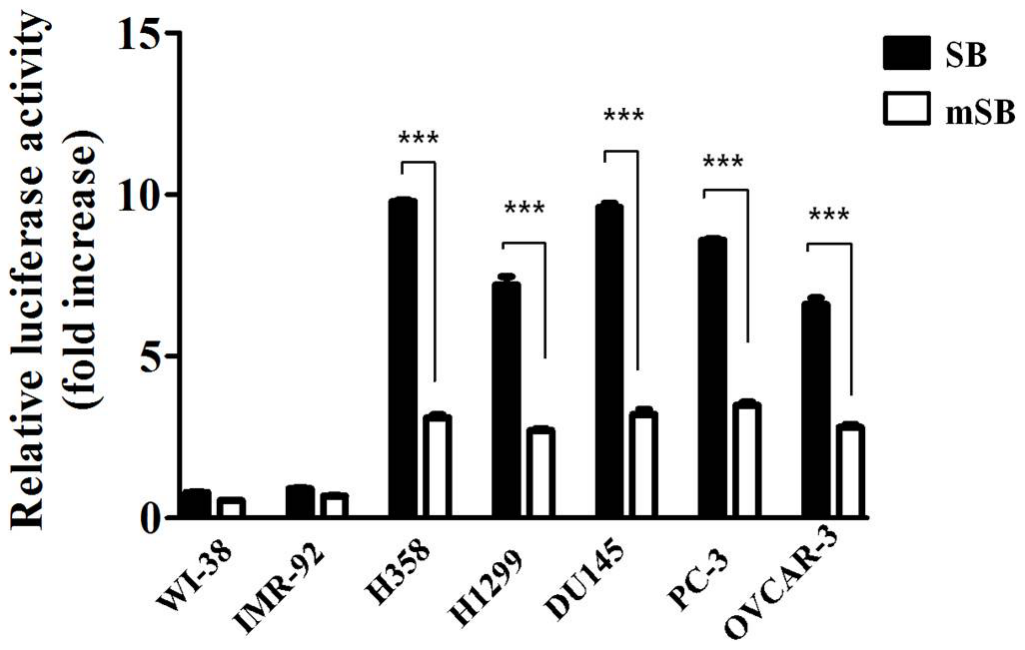
Long-lasting hTERT-mediated transcriptional activity in various types of cancer cells. Luciferase assays were used to determine the promoter activities of the luciferase expressing plasmid pT-hTp-Con following co-transfection with either the active helper plasmid (pCMV-SB) or the inactive helper plasmid (pCMV-mSB) in H358, H1299, PC3, DU145, OVCAR3, WI-38, and IMR-90 cells. All cancer cell lines that contained the SB-based gene delivery system, under the control of the hTERT promoter and the SV40 enhancer, sustained relatively higher levels of long-term luciferase activity than the normal fibroblasts 10 days after transfection. Relative luciferase activity was standardized to the transfection of the control pGL3-promotor plasmid. Lung cancer cell lines (H358 and H1299); prostate cancer cell lines (PC3 and DU145); ovarian cancer cell line (OVCAR3); fibroblast cell lines (WI-38 and IMR-90). The results are shown as the mean ± SD from three independent experiments. * P<0.05, ** P<0.01, and *** P<0.001.

Successful gene therapy depends on the long-term sustainability of therapeutic gene expression to cure or slow down the progression of cancer development. Therefore, we next examined whether the SB delivery system modified with the hTERT promoter and the SV40 enhancer sustained long-lasting therapeutic gene expression in various types of cancer cell lines. Because the transposase-producing helper plasmid is essential for the insertion of SB into the host genomes, we co-transfected normal fibroblast and cancer cell lines with a luciferase expressing version of the pT-hTp-Con plasmid along with either the active helper plasmid (pCMV-SB) or the inactive helper plasmid (pCMV-mSB). All cancer cell lines sustained relatively higher levels of long-term luciferase activity using the SB delivery system under control of the hTERT promoter and the SV40 enhancer compared to the normal fibroblasts; there was no change in the two normal fibroblast cell lines 10 days after transfection (Fig. 4). These findings suggest that the modified SB delivery system is a promising way to enhance the therapeutic efficiency when long-term expression of the therapeutic products is required.

### Modified SB delivery system increases tumor cell specific cytotoxicity

**Figure 5:**
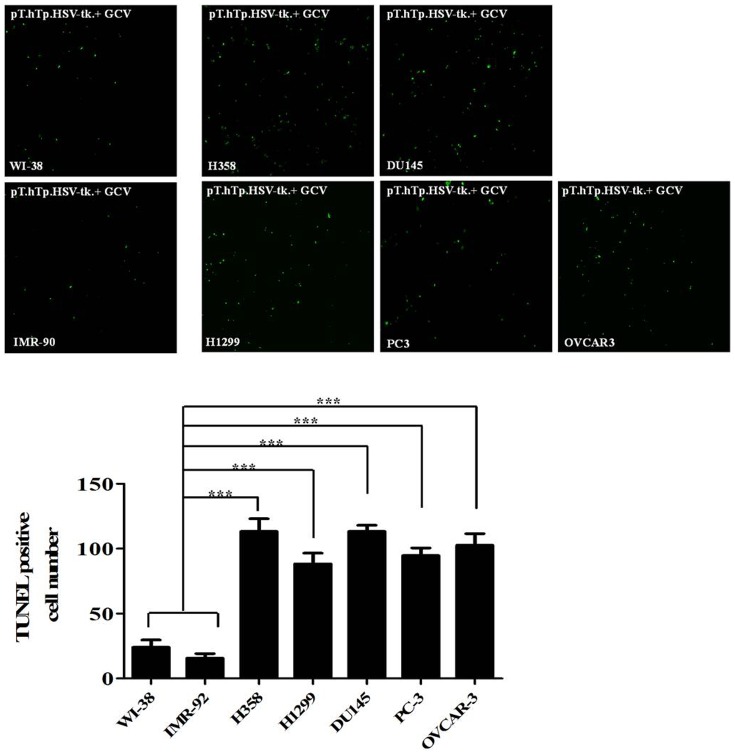
Cancer cell specific effect of the SB-based gene delivery system with the hTERT promoter. The levels of cell apoptosis were assessed using the TUNEL assay after transfection of the SB system (pT.hTp.HSV-tk.Con with active helper plasmid) and following administration of 50 µg/mL GCV at 3 day post-transfection. TUNEL-positive cells were characterized by densely stained circular bodies that represent the fragmented DNA of apoptotic cells. SB transfected cancer cells showed a significantly increased number of TUNEL-positive cells compared to normal fibroblasts in the presence of 50 µg/mL GCV. Lung cancer cell lines (H358 and H1299); prostate cancer cell lines (PC3 and DU145); ovarian cancer cell line (OVCAR3); fibroblast cell lines (WI-38 and IMR-90). The results are shown as the mean ± SD from three independent experiments. * P<0.05, ** P<0.01, and *** P<0.001.

**Figure 6:**
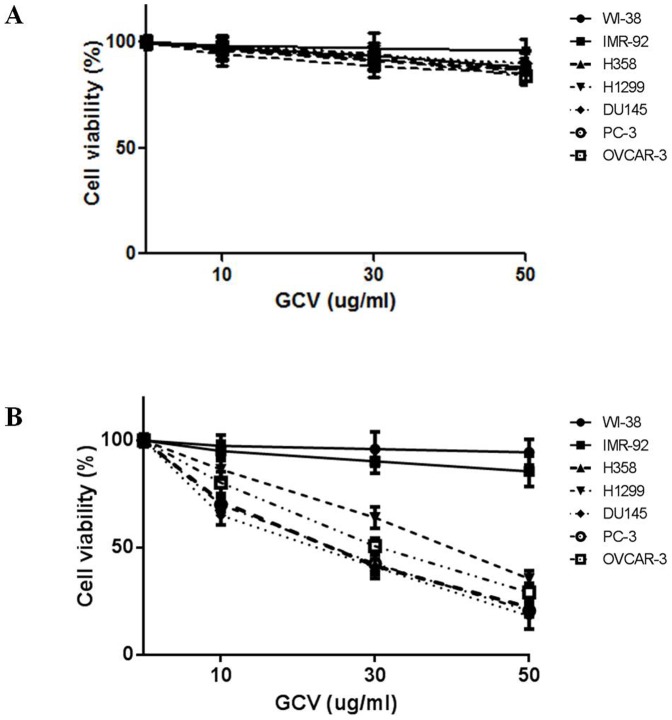
Cancer cell specific effect of the SB-based gene delivery system with the hTERT promoter on cell viability. Cell viability was assessed using the MTT with or without transfection of the SB system (pT.hTp.HSV-tk.Con with active helper plasmid) following administration of 10, 30, or 50 µg/mL GCV at 3 day post-transfection. GCV alone had negligible effect on effect on the cell viability (A), whereas transfection of the SB system (pT.hTp.HSV-tk.Con with active helper plasmid) following administration of GCV significantly decreased the cancer cell viability in a dose-dependent manner (B). Lung cancer cell lines (H358 and H1299); prostate cancer cell lines (PC3 and DU145); ovarian cancer cell line (OVCAR3); fibroblast cell lines (WI-38 and IMR-90). The results are shown as the mean ± SD from three independent experiments. * P<0.05, ** P<0.01, and *** P<0.001.

**Figure 7:**
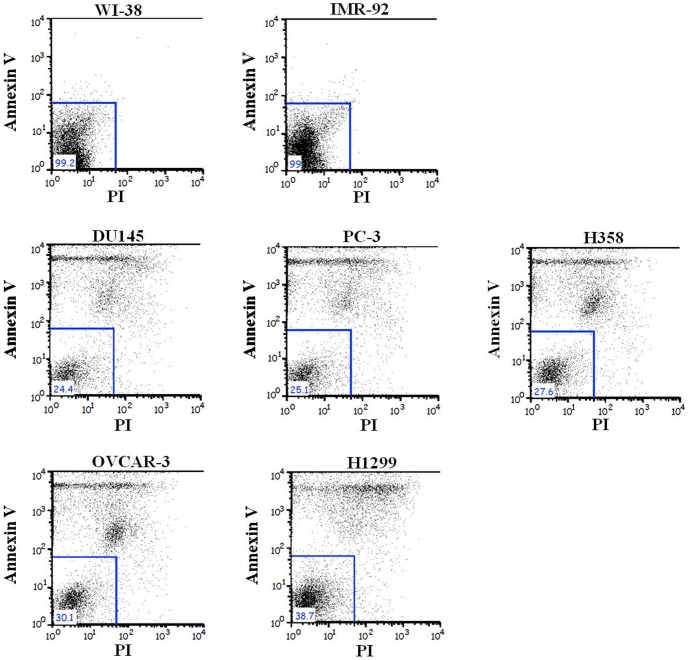
Flow cytometry analysis of the SB-based gene delivery system with the hTERT promoter on cancer cell apoptosis. Cancer cell lines and normal fibroblasts were transfected with the SB system (pT.hTp.HSV-tk.Con with active helper plasmid). Cells were then challenged with 50 µg/mL GCV, followed by Annexin V-FITC/PI staining and FACS analysis at 3 day post-transfection. Lung cancer cell lines (H358 and H1299); prostate cancer cell lines (PC3 and DU145); ovarian cancer cell line (OVCAR3); fibroblast cell lines (WI-38 and IMR-90).

**Figure 8:**
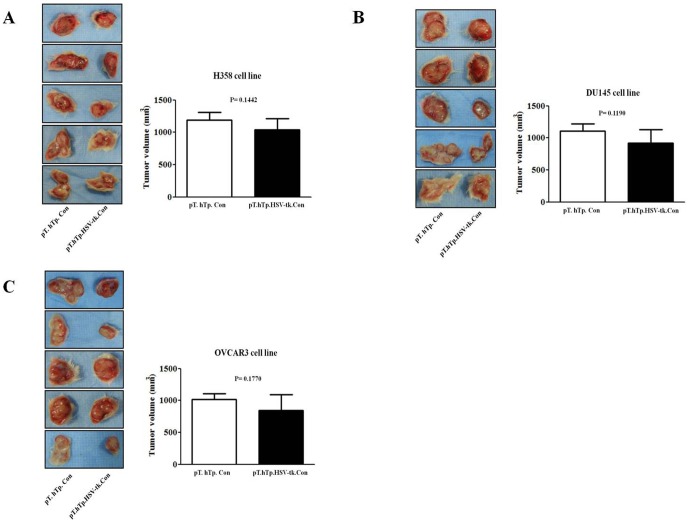
The effect of modified SB system on the tumor growth in vivo. Lung cancer cells (H358) (A), prostate cancer cell line (DU-145) (B), and ovarian cancer cells (OVCAR3) (C) were harvested by trypsinization, and 1×10^5^ viable cells (as determined by trypan blue exclusion) in a total volume of 200 µl were injected subcutaneously. Two days following tumor seeding, animals were intravenously injected via tail veins with 100 mg/kg gancyclovir (GCV) along with either co-transfection of the empty plasmid (pT. hTp. Con) with the active helper plasmid (pCMV-SB) or co-transfection of the SB system (pT.hTp.HSV-tk.Con) with the active helper plasmid (pCMV-SB). Mice were sacrificed 28 days after tumor injection, and the effect of modified SB system on tumor growth was evaluated by measuring tumor size.

Successful cancer treatment using gene therapy largely depends on the specific delivery of the therapeutic product into the appropriate target cancer cells. Gene-directed enzyme prodrug therapy (GDEPT) is a promising alternative to conventional chemotherapy; GDEPT minimizes the systemic toxicities of conventional chemotherapy drugs by introducing catalytic enzymes into tumor cells; these enzymes then convert low- or non-toxic prodrugs into toxic metabolites [1]. After genetically modifying the tumor cells to express the catalytic enzymes, systemic administration of the prodrug, which is locally converted by the enzyme into cytotoxic metabolites, leads to the selective killing of tumor cells. To determine the tumor cell specific toxic effect of our SB-based gene delivery system, cancer cells and normal fibroblasts were co-transfected with the modified SB system (pT.hTp.HSV-tk.Con) along with the active helper plasmid (pCMV-SB) following the administration of 50 µg/mL gancyclovir (GCV). We then performed TUNEL assays on cells 10 days after transfection to identify the apoptotic cells, which are characterized by the presence of densely stained circular bodies that represent the fragmented DNA of apoptotic cells. As shown in Fig. 5, the TUNEL assays demonstrated that the modified SB transfected cancer cells showed a significantly increased death rate compared to the normal fibroblasts in the presence of 50 µg/mL GCV. To further confirm this tumor specific cell death by the SB-based gene delivery system, a cell viability assay was used to evaluate the cancer cell specific cytotoxicity in the normal fibroblasts and cancer cell lines treated with GCV in a dose-dependent manner. GCV alone had negligible effect on the cell viability (Fig. 6A), whereas transfection of the modified SB (pT.hTp.HSV-tk.Con) system following administration of GCV significantly decreased cancer cell viability in a dose-dependent manner (Fig. 6B). Additionally, cell death was also determined by flow cytometry to identify cells that were stained with both Annexin V/FITC and propidium iodide (PI). The data from the TUNEL and cell viability assays were consistent with our flow cytometry results (Fig. 7). These results suggest that our SB-based gene delivery system mediated tumor cell specific therapeutic gene delivery, which in turn induced tumor-specific apoptosis. Our in vitro data suggested that the modified SB transfected cancer cells showed a significantly increased death rate compared to the normal fibroblasts. Therefore, we next investigated whether tumor growth could be suppressed by modified SB delivery system in vivo. In some experimental groups, tumor growth was successfully suppressed by modified SB delivery system, while the other groups did not show an apparent anti-tumor effect (Fig. 8).

## Discussion

Many commonly used chemotherapy drugs lack tumor specificity, and the doses required to reach therapeutic levels in the tumor are often toxic to the surrounding normal tissues. Therefore, prodrug-activating systems that include suicide gene therapy are promising alternatives to conventional chemotherapy; these systems minimize the systemic toxicities of conventional chemotherapy drugs. For clinical application, the suicide genes need to satisfy the following criteria: a) these genes must be either not expressed or present at extremely low levels in the host; and b) the gene must have a high catalytic activity to achieve a sufficient level of the toxic metabolite in the tumors [36]. Ideally, tumor-targeted prodrugs must meet the following criteria: a) the prodrug must be less toxic or non-toxic prior to activation (cleavage) by the suicide gene product; b) the prodrug must have a selective binding affinity for the transfected suicide gene product; and c) an active metabolite of the prodrug must have an extended half-life so that the treatment dose can be reduced [36].

Of the suicide gene/prodrug therapy systems, by far the combination of HSV-TK and GCV is the most frequently used in cancer gene therapy. The HSV-TK gene is transfected into the tumor cells to convert the systemically administered non-toxic prodrug GCV into a toxic metabolite [37]. HSV-TK catalyzes GCV into monophosphorylated GCV (GCV-MP), which is then converted into the toxic triphosphate form of GCV (deoxythymidine triphosphate). This triphosphate metabolite is an analog of purine; incorporation of this analog during DNA synthesis inhibits DNA polymerase, leading to the observed toxicity [38]. Therefore, this combination method has been successfully applied to many clinical areas, such as gene therapy for cancer treatment [6], as an efficient tool for controlling graft-versus-host disease (GVHD) [7], and as positron emission tomography (PET) reporter probes [39]. However, this combination system is not effective enough to eradicate malignant tumors due to the poor transfection efficiency of the HSV-TK gene, resulting in significantly lower expression levels of the gene product *in vitro* and *in vivo* [40]; poor expression of the HSV-TK gene requires that higher doses GCV are used during treatment. High doses of GCV appear to be associated with hematologic toxicities, such as leucopenia and thrombocytopenia, renal toxicity, and other adverse side effects [41]. These disadvantages have greatly limited the clinical application of the HSV-TK/GCV system. However, it is generally thought that these limitations are associated with the poor transfection efficiency of the gene delivery systems used in these experiments rather than a failure of the combination gene therapy using HSV-TK and GCV [42].

Several studies have focused on increasing the transfection efficiency and the expression level of the HSV-TK gene to improve the therapeutic potential of the HSV-TK/GCV combination system. Many transfection methods have been attempted to improve the transfection efficiency, but most of the observed effects did not meet the clinical requirements, such as safe, non-immunogenic, easy to produce, target specific, and long-lasting expression in tumor cells. The SB transposon-based system is an attractive, non-viral alternative to the previously used viral delivery systems. SB may be less immunogenic than viral vector systems due to lack of viral sequences [43]. The SB-based gene delivery system can stably integrate into the host cell's genome to produce the suicide gene product over the cell's lifetime [44]. SB-mediated transposition has been shown to occur in a variety of cell culture systems including zebrafish [45], mouse embryo [46], mouse lung and liver [47]–[49], and human primary blood lymphocytes [50]. However, when compared to the viral vectors, the non-viral SB-based gene delivery system had limited therapeutic efficacy due to the lack of long-lasting gene expression and tumor cell specific gene transfer ability. This limitation can be overcome through the addition of the hTERT promoter and the SV40 enhancer to the SB transposon. hTERT, the catalytic subunit of telomerase, is highly expressed in embryonic stem cells, is progressively down-regulated during differentiation, and is silenced in fully differentiated somatic cells. hTERT is frequently reactivated in approximately 90% of immortalized human cells and cancer cells of various origins [26]–[28]. Moreover, its powerful promoter is essential to achieve long-term stable expression of a therapeutic gene. The SV40 enhancer has been extensively used to improve the activity of promoters [32]. Thus, to increase the promoter strength while maintaining tissue specificity, we constructed a recombinant SV40 enhancer containing the tumor-specific hTERT promoter.

Our in vitro data suggested that the modified SB transfected cancer cells showed a significantly increased death rate compared to the normal fibroblasts. Therefore, we next investigated whether tumor growth could be suppressed by modified SB delivery system in vivo. In some experimental groups, tumor growth was successfully suppressed by modified SB delivery system, while the other groups did not show an apparent anti-tumor effect (Fig. 8). These in vivo variations among the experimental subjects possibly due to the following: 1) the lower in vivo transfection efficiency of SB plasmid can be related directly to the lower anti-tumor effect of modified SB delivery system in vivo; 2) One of the critical parameters for a successful expression of systemic administration of plasmid DNA is the volume of DNA solution administered. Previous study demonstrated that plasmid delivery to rodent by the tail vein is effective as long as the volume of injected DNA solution is adjusted to 7–8% of body weight [51]; 3) rapid injection (less than 10 s) is also critical parameters for gene delivery with highest level of gene expression [51].

In this study, the SB-based delivery system, under the control of the hTERT promoter with the SV40 enhancer, was used to achieve successful target specific transfer of the HSV-TK gene into multiple types of cancer. Our SB-based system induced HSV-TK gene expression exclusively in cancer cells due to the cancer cell specific hTERT promoter activity, and therefore only the cancer cells were susceptible to the administration of the GCV prodrug. In the presence of GCV, the SB transfected cancer cells showed a significantly increased death rate compared to the normal fibroblasts. The viability of the SB transfected cancer cells was significantly lower than that of the normal fibroblasts following GCV treatment; this result was dose-dependent. These data suggest that our modified SB-based gene delivery system can be used as a safe and efficient tool for stable HSV-TK gene transfer and long-term expression. However, several critical questions still need to be answered before a clinical trial of SB mediated therapeutic gene delivery can commence. First, it is still possible that SB functions as an insertion mutagen that disrupts the structure of the host gene where it resides, resulting in tumor formation. Second, SB and its associated carriers, such as PEI, can instigate an inflammatory response. Finally, our understanding of the interactions between SB and intracellular molecules is still rudimentary.
